# Potential risk of certain cancers among patients with Periodontitis: a supplementary meta-analysis of a large-scale population

**DOI:** 10.7150/ijms.46812

**Published:** 2020-09-12

**Authors:** Haozhen Ma, Jianmao Zheng, Xiaolan Li

**Affiliations:** 1 Hospital of Stomatology, Sun Yat-sen University, Guangdong Provincial Key Laboratory of Stomatology, 510055 Guangzhou/PR. China.; 2 Guanghua School of Stomatology, Sun Yat-sen University, 510055 Guangzhou/PR. China.

**Keywords:** Esophageal cancer, prostate cancer, hematological malignancy, melanoma of the skin, periodontitis, meta-analysis

## Abstract

**Background:** Some studies have reported biological linkages between periodontitis and esophageal cancer, prostate cancer, kidney cancer, hematological malignancy, and melanoma of the skin. This meta-analysis aimed to assess the relationship between periodontitis and the aforementioned five cancers.

**Methods:** Eligible studies on the association between periodontitis and the aforementioned five kinds of cancers were retrieved. The statistical analysis was conducted using Stata 12.0.

**Results:** Ten articles (more than 100,000 samples for most cancers) were included. With statistical significance, participants with periodontitis might have enhanced risks of esophageal cancer (HR = 1.79, 95% CI: 1.15-2.79), prostate cancer (HR = 1.20, 95% CI: 1.09-1.31), hematological malignancy (HR = 1.19, 95% CI: 1.09-1.29), and melanoma of skin (HR = 1.21, 95% CI: 1.03-1.42), compared with those without periodontitis. However, the evidence regarding the correlation between periodontitis and the susceptibility to kidney cancer was lacking (HR=1.30, 95% CI: 0.96-1.76).

**Conclusions:** The present meta-analysis revealed a potential link between periodontitis and esophageal cancer, prostate cancer, hematological malignancy, and melanoma of the skin. However, multi-center studies with large sample sizes and multivariable adjustments are still needed to support the conclusion.

## Introduction

Periodontitis is an inflammatory disease that destroys periodontal tissues (periodontal ligament and alveolar bone). About 10%-15% of the global population suffers from substantial tooth loss caused by severe periodontitis 1. However, the impact of periodontitis is not confined to oral health. Multiple meta-analyses revealed that participants with periodontitis tended to develop malignant tumors, including oral 2, head and neck 3, lung 4, breast 5, pancreatic 6, and so forth.

However, the correlation between periodontitis and other types of cancers, including esophageal cancer, prostate cancer, kidney cancer, hematological malignancy (HM), and melanoma of the skin, remains unclear. The aforementioned cancers are characterized by high morbidity and fatality rate. According to the GLOBOCAN 2018 database 7, esophageal cancer was the sixth fatal cancer. Prostate cancer was the second most common cancer among men, while renal cancer and melanoma of the skin were the top 20 common cancers. In the USA, HM was the fourth most susceptible cancer, accounting for 9.7% of all cancers 8. Therefore, the risk factors of these cancers are needed to be explored to reduce their risk.

Recently, mounting evidence suggested that periodontitis was potentially associated with esophageal cancer, prostate cancer, kidney cancer, HM, and melanoma of the skin, of which esophageal and prostate cancer were the most studied. Periodontal pathogens with their DNA or virulence factors were detected in esophageal cancer tissues, including *Porphyromonas gingivalis* (*P.gingivalis*) 9*, Fusobacterium nucleatum* (*F. nucleatum*) 10 and *Treponema denticola* (*T. denticola*) 11. Moreover, *P.gingivalis* was found to be correlated with the differentiation, metastasis and clinical stage of esophageal cancer. A high level of immune globulins against *P.gingivalis* was related to the poor prognosis of esophageal cancer 12. As for prostate cancer, scholars found the same pathogens in prostate secretions and dental plaque, including *P. gingivalis* and *T. denticola* 13. Furthermore, prostate-specific antigen (PSA, a marker of inflammation or malignancy of the prostate) level was found to be associated with clinical attachment level 14 and periodontal treatment 15. As for renal cancer, HM, and melanoma of the skin, epidemiological evidences indicated that patients with periodontitis may have enhanced susceptibility to these cancers 16-21. Besides, associations between periodontitis and some rare subtypes of HM, such as chronic lymphocytic leukemia 22 and lymphoplasmacytic lymphoma-Waldenström macroglobulinemia 23, were discovered.

Although there were biologically possible links between periodontitis and the aforementioned five types of malignant tumors, epidemiological researches revealed conflicting results. Due to the lack of statistical effectiveness of a single epidemiological study, this meta-analysis was conducted to provide the latest evidence on the relationship between periodontitis and the aforementioned five cancers.

## Methods

### Study retrieval and selection

Articles published up to August 2020 on the association between periodontitis and the aforementioned five kinds of cancers, were retrieved from PubMed, Web of Science, Embase, and Cochrane Library. The key words were as follows: (1) “esophageal cancer” OR “esophagus tumor” OR “prostate cancer” OR “prostate tumor” OR “kidney cancer” OR “kidney tumor” OR “hematologic malignancy” OR “hematologic cancer” OR “hematologic neoplasm” OR “leukemia” OR “lymphoma” OR “multiple myeloma” OR “melanoma” AND (2) “periodontitis” OR “attachment loss” OR “probing depth” OR “alveolar crestal height”. Meanwhile, the references of related studies were also reviewed.

A total of 375 records were obtained, and studies were selected using the following inclusion criteria: (1) studies on human participants; (2) exposure of interest being patients with periodontitis and the control being healthy participants without periodontitis; (3) result of concern being the incidence of esophageal cancer, prostate cancer, kidney cancer, HM, or melanoma of the skin; (4) studies designed as cohort studies, with the full text available; and (5) adjusted hazard ratio (HR) and 95% confidence intervals (95% CI) provided and adjusted confounders reported.

**Figure 1:**
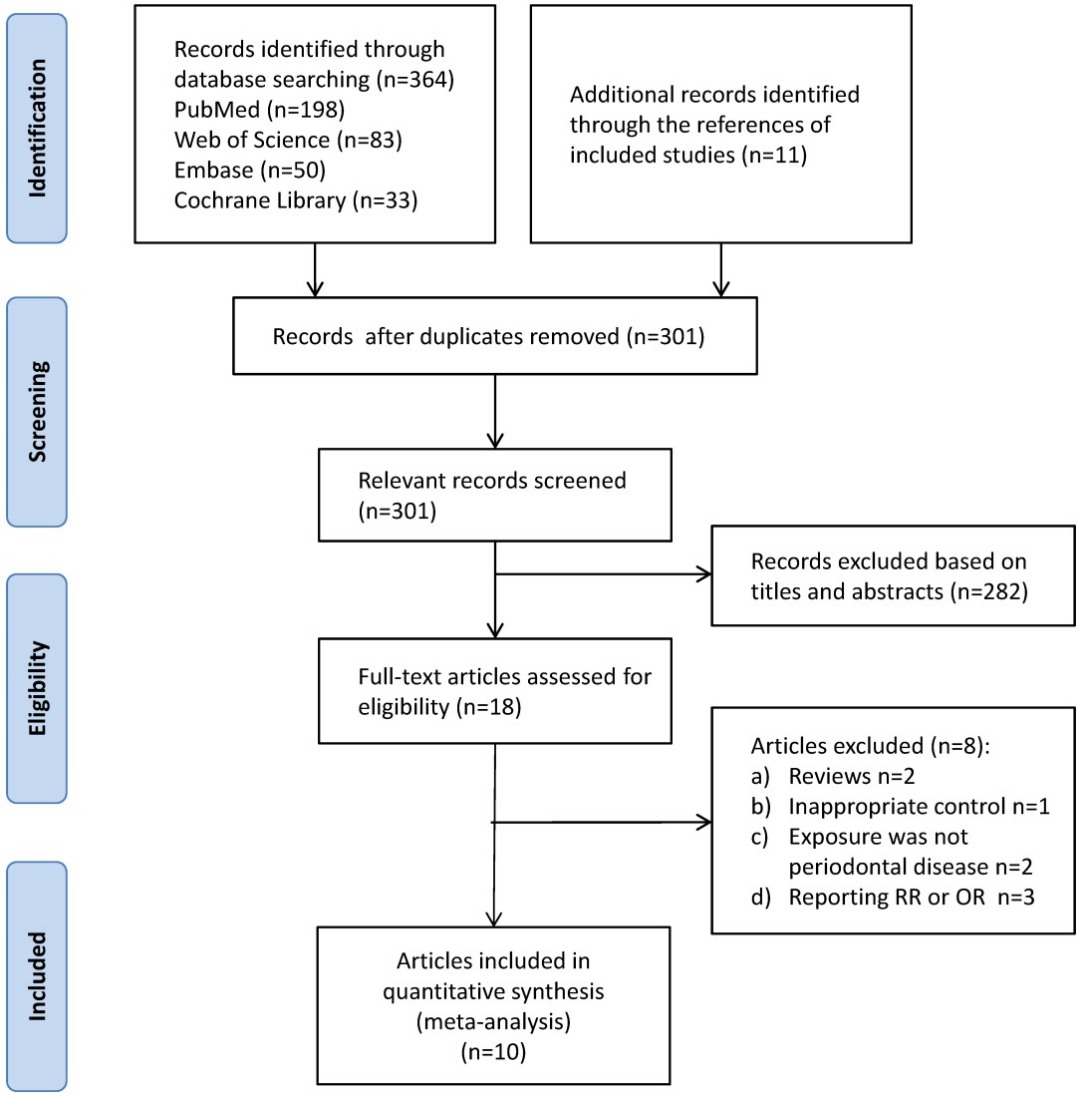
Process of retrieval and selection.

The retrieval and selection were done by two independent authors. Ten articles (referring to 8 studies) were finally included. The detailed process of searching and selection was illustrated in **Figure 1.**

### Ascertainment of periodontitis and cancers

#### Ascertainment of periodontitis (precise)

- A diagnosis code for periodontitis (ICD-9-CM: 523.0-523.5) 21, 24;
- At least two teeth with interproximal (between teeth) attachment loss ≥4 mm or at least two teeth with interproximal pocket depth ≥5 mm (guidelines of the CDC and the American Academy of Periodontology, CDC/AAP) 25;
- Whole mouth mean alveolar crestal height (ACH) ≥2 mm, or at least one site ACH ≥4mm (ACH was defined as the distance in millimeters from the cemento-enamel junction to the alveolarcrest) 20;
- >2 interproximal sites with attachment loss (AL) >3 mm, and >2 interproximal sites with probing depth (PD)>4 mm (not on same tooth) or one site with PD>5 mm 26;
- >10% of examined sites(six sites on all teeth) having AL>3 mm 26;

#### Ascertainment of periodontitis (imprecise)

- Self-reported teeth mobility (at least half of the teeth had mobility) 27.

#### Ascertainment of the 5 cancers

- Diagnosis codes for the 5 cancers, such as ICD-9-CM 150 (esophageal cancer), ICD-10-CM C61 (prostate cancer) 18, 21, 24, 25, 28;
- Records on cancer registries or death certificates where cancer was the underlying cause of death 27, 29.

Detailed criteria of classification of periodontitis severity were shown in **Table 1.**

### Information extraction and quality evaluation

The following information was obtained from 8 involved studies (referring to 10 articles): author, published date, origin of participants, study design, mean follow-up period, mean age, sex, sample size, ascertainment of periodontitis and cancers, severity of periodontitis, cancer types, adjusted covariates and information for quality assessment. Besides, two authors independently evaluated the quality of eligible literatures using the Newcastle-Ottawa scale (NOS). The maximum score could reach 9 points for each study. The specific NOS score was proportional to study quality. NOS scores were divided into three levels: a score of 0-3, relatively low quality; a score of 4-6, moderate quality; and a score of 7-9, relatively high quality. **Tables 2 and 3** illustrated the extracted data and NOS scores. The risk of bias was evaluated by two authors based on a scale in Review Manager 5.2, which included evaluation for cohort design, blinding, missing results, incomplete reporting and so forth. The result was shown in **Figure S1** and **Figure S2** in the Supplementary File.

### Statistical analysis

We employed the inverse-variance model in Stata 12.0 to obtain overall estimates. HR worked as an indicator to evaluate the correlation between periodontitis and cancers. The heterogeneity was evaluated using *I*^2^ metric values and their 95% CIs 30. The 95% CIs of *I^2^* were calculated based on the classical formulas proposed by Higgins 30. Random- or fixed-effects models were selected according to heterogeneity evaluation. However, if the number of studies on each type of cancer was less than 10, overall estimates were calculated using random‐effects models. Publication bias assessments were conducted through funnel plots. However, funnel plots could not be applied if fewer than 10 studies were included, according to the Cochrane handbook 31. Stratified by different characteristics of the involved studies, subgroup analyses were used to explore whether the study characteristics influenced the pooled results. Sensitivity analyses were carried out for the evaluations of results' robustness by individually omitting one study at a time. For some types of cancers with few related studies, the sensitivity and subgroup analyses might be waived.

## Results

The included studies were pooled and analyzed by different cancers, as illustrated in **Table 4.** Overall estimates were calculated employing random‐effects models because of the insufficient number of studies (<10 studies) on each type of cancer. With statistical significance, participants with periodontitis might have enhanced risks of esophageal cancer, prostate cancer, hematological malignancy, and melanoma of the skin, compared with those without periodontitis. However, the evidence regarding the correlation between periodontitis and the susceptibility to kidney cancer was lacking.

### Esophageal cancer

**Figure 2:**
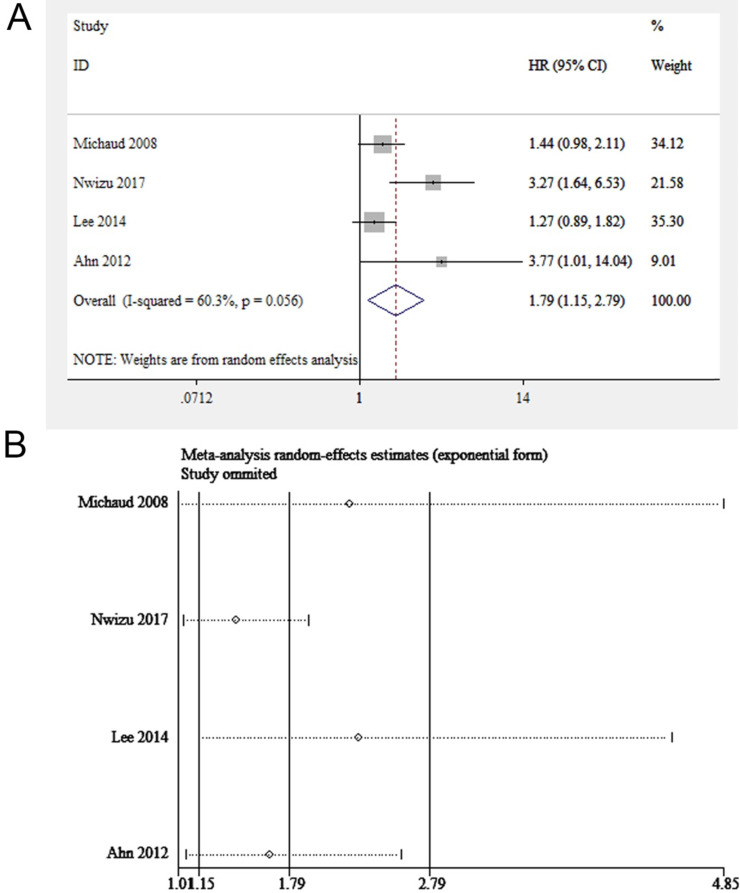
(**A**) Forest plot and (**B**) sensitivity analysis of studies on periodontitis and esophageal cancer.

Among all the included studies, four focused on periodontitis and the risk of esophageal cancer (845,258 participants in total) 17, 18, 24, 25. The results showed that the exposure to periodontitis might enhance the susceptibility to esophageal carcinoma (HR = 1.79, 95% CI: 1.15-2.79, *P* = 0.009, *I^2^* = 60%, P for heterogeneity = 0.056, **Figure 2A**). As shown in **Figure 2B**, the pooled results remained robust when individually leaving out one study at a time. Funnel plot was not applied because of the small number of included studies (<10 studies).

Since significant heterogeneity was discovered, a subgroup analysis stratified by sex, age, research quality and adjusted covariates was performed to find out the causes for heterogeneity (**Table 5**). When stratified by research quality, the value of *I^2^* decreased significantly, indicating that the main source of heterogeneity was study quality. As for sex, the heterogeneity of the male group was not significant while that of the female group was obvious, suggesting that sex contributed partly to heterogeneity. When pooling the studies with the mean age of participants ≥50, we found periodontitis was potentially linked to esophageal carcinoma (HR=2.12, 95%CI: 1.01-4.45). When pooling studies with adjusted covariates of smoking or socio-economic status, we discovered a significant correlation between periodontitis and esophageal carcinoma. None of the four studies had a classification of periodontitis severity; therefore, no subgroup analysis was performed on periodontitis severity.

### Prostate cancer

**Figure 3:**
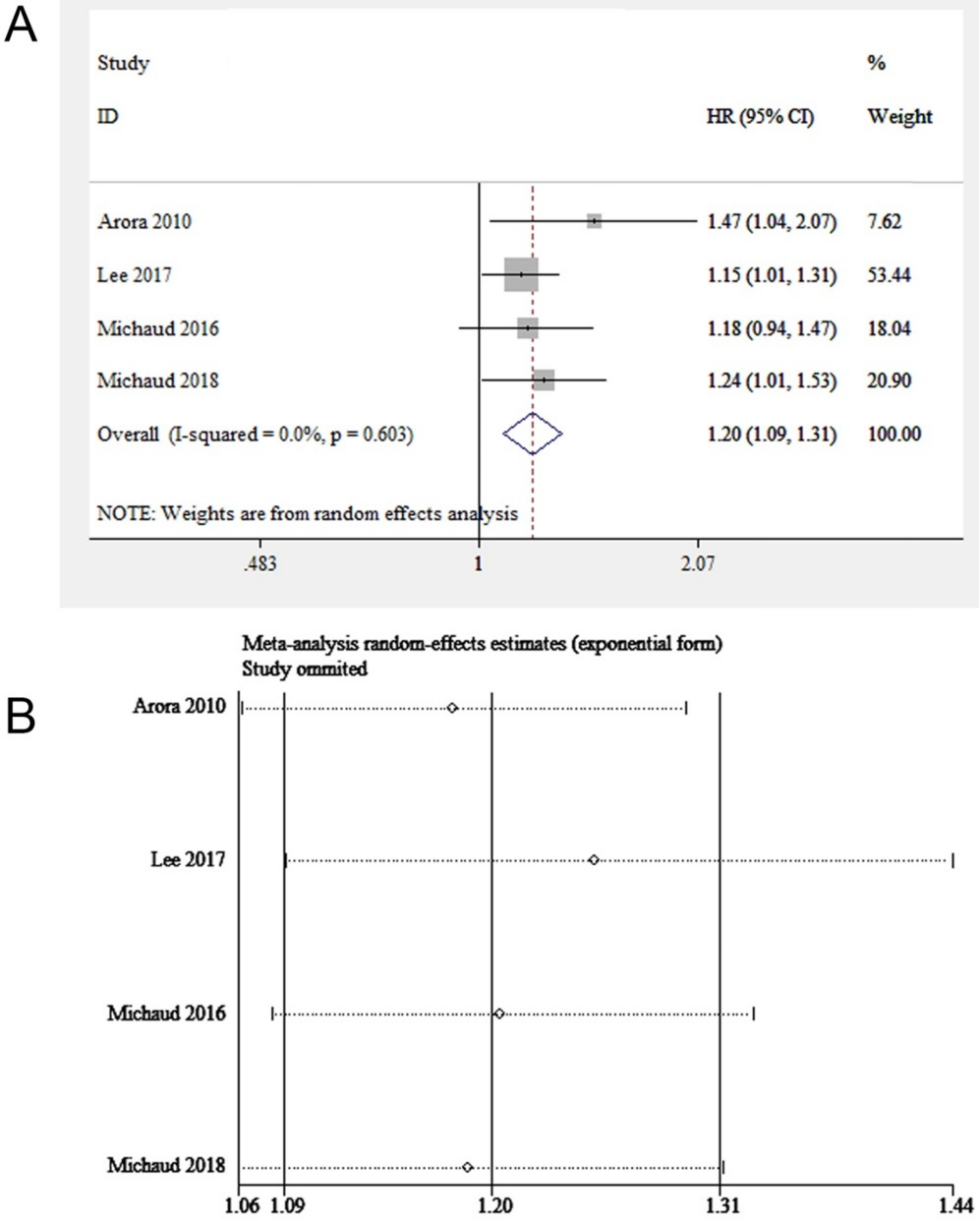
(**A**) Forest plot and (**B**) sensitivity analysis of studies on periodontitis and prostate cancer.

Four eligible studies (involving 229,256 participants) focused on periodontitis and prostate cancer 16, 26-28. We found the exposure to periodontitis was potentially linked to prostate cancer (HR = 1.20, 95% CI: 1.09-1.31, **Figure 3A**). The sensitivity analysis illustrated the robustness of the results (**Figure 3B**). The funnel plot was not used due to the insufficient number of included studies (< 10 studies).

The subgroup analysis stratified by severity of periodontitis, age, sample size and adjusted covariates was illustrated in **Table 6.** Among the four studies included, two studies investigated the association between periodontitis of different severity and prostate cancer 26, 27, which were pooled in this subgroup analysis. Mild/Moderate periodontitis might not be related to the enhanced susceptibility to prostate carcinoma, while severe periodontitis was responsible for the enhanced risk of prostatic carcinoma. A correlation between periodontitis and prostate cancer was found when adjusting smoking or socio-economic status. Regarding sample size, a higher risk estimate was obtained from studies with small sample sizes compared to studies with large sample sizes.

### Kidney cancer

**Figure 4:**
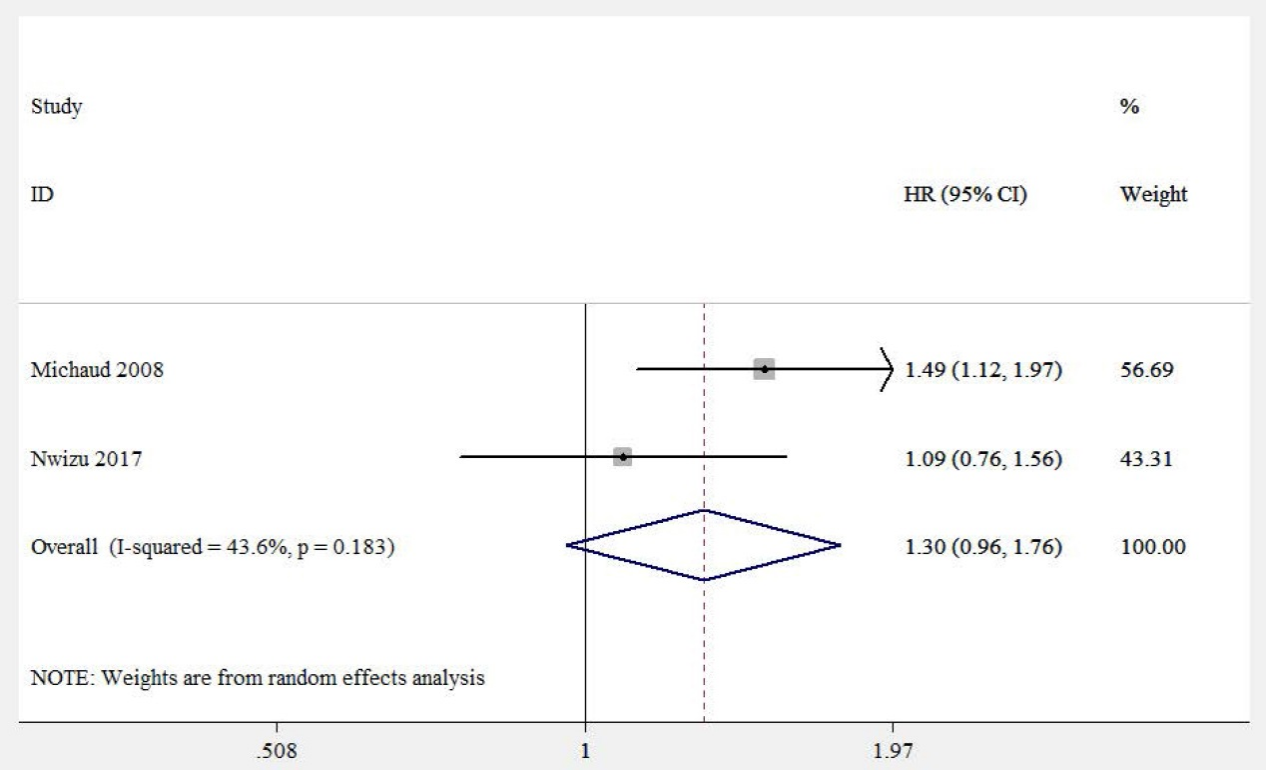
Forest plot of studies on periodontitis and kidney cancer.

Two eligible studies (involving 114,244 participants) focused on correlation between periodontitis and kidney cancer risk 17, 18. Both of them adjusted important confounders, such as age, race, smoking status, drinking status, fruit and vegetable intake, frequency of exercise and history of diabetes. However, no evident correlation between periodontitis and kidney cancer was found (HR=1.30, 95%CI: 0.96-1.76, **Figure 4**).

### Hematological malignancy

**Figure 5:**
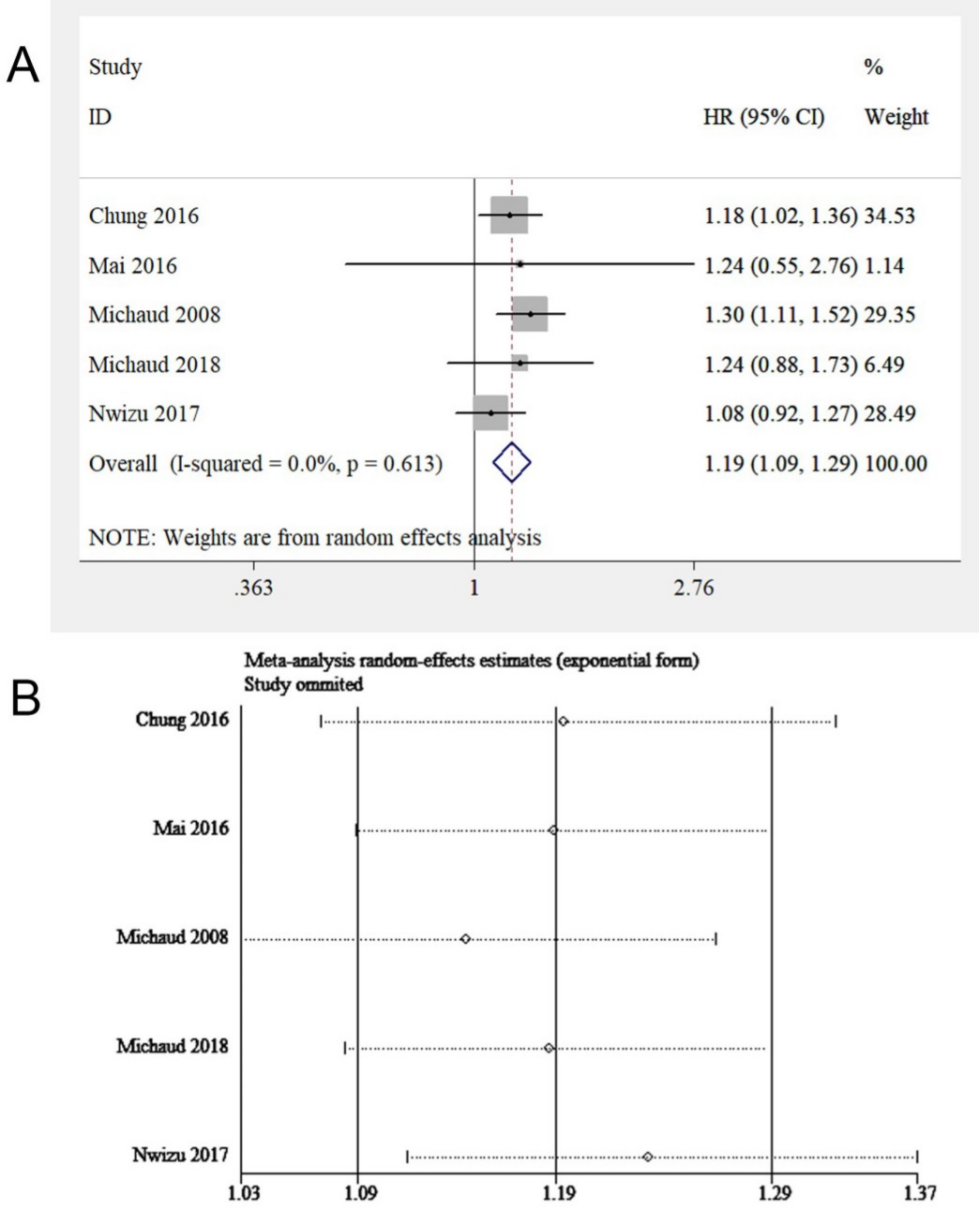
(**A**) Forest plot and (**B**) sensitivity analysis of studies on periodontitis and HM.

Epidemiological studies concerning periodontitis and cancers always combined the cases of leukemia, lymphoma, and multiple myeloma (MM), collectively known as HM. Thus, this study evaluated the relationship between periodontitis and HM, while the respective correlation between periodontitis and leukemia, lymphoma, or MM was assessed in the subgroup analysis. Five eligible studies 17, 18, 20, 21, 26 were involved, with 201,917 samples in total. We found periodontitis was potentially associated with HM risk (HR = 1.19, 95% CI: 1.09-1.29, **Figure 5A**). Sensitivity analysis illustrated the homogeneousness and robustness of the results (**Figure 5B**). Because of insufficient studies involved (<10 studies), the funnel plot was not applied to evaluate publication bias.

Subgroup analysis was conducted stratified by periodontitis severity, adjusted covariates, age, sex, follow-up duration, sample size, and HM sub-types (**Table 7**). A correlation between periodontitis and HM was observed among participants with mean age <60 years and male participants. When pooling studies with adjustments on smoking status or socioeconomic factors, a link between periodontitis and HM was discovered. Besides, an association between periodontitis and HM was observed in non-smokers. For the follow-up years, the longer the duration, the higher the HM risk for patients with periodontitis (≤10 years: HR=1.13, 95% CI: 1.02-1.26; >10 years: HR = 1.29, 95% CI: 1.12-1.48). For HM sub-types, periodontitis enhanced the susceptibility to non-Hodgkin's lymphoma (NHL) (HR = 1.20, 95% CI: 1.01-1.43), but the evidence that periodontitis could increase the risk of leukemia and MM was lacking.

### Melanoma of the skin

**Figure 6:**
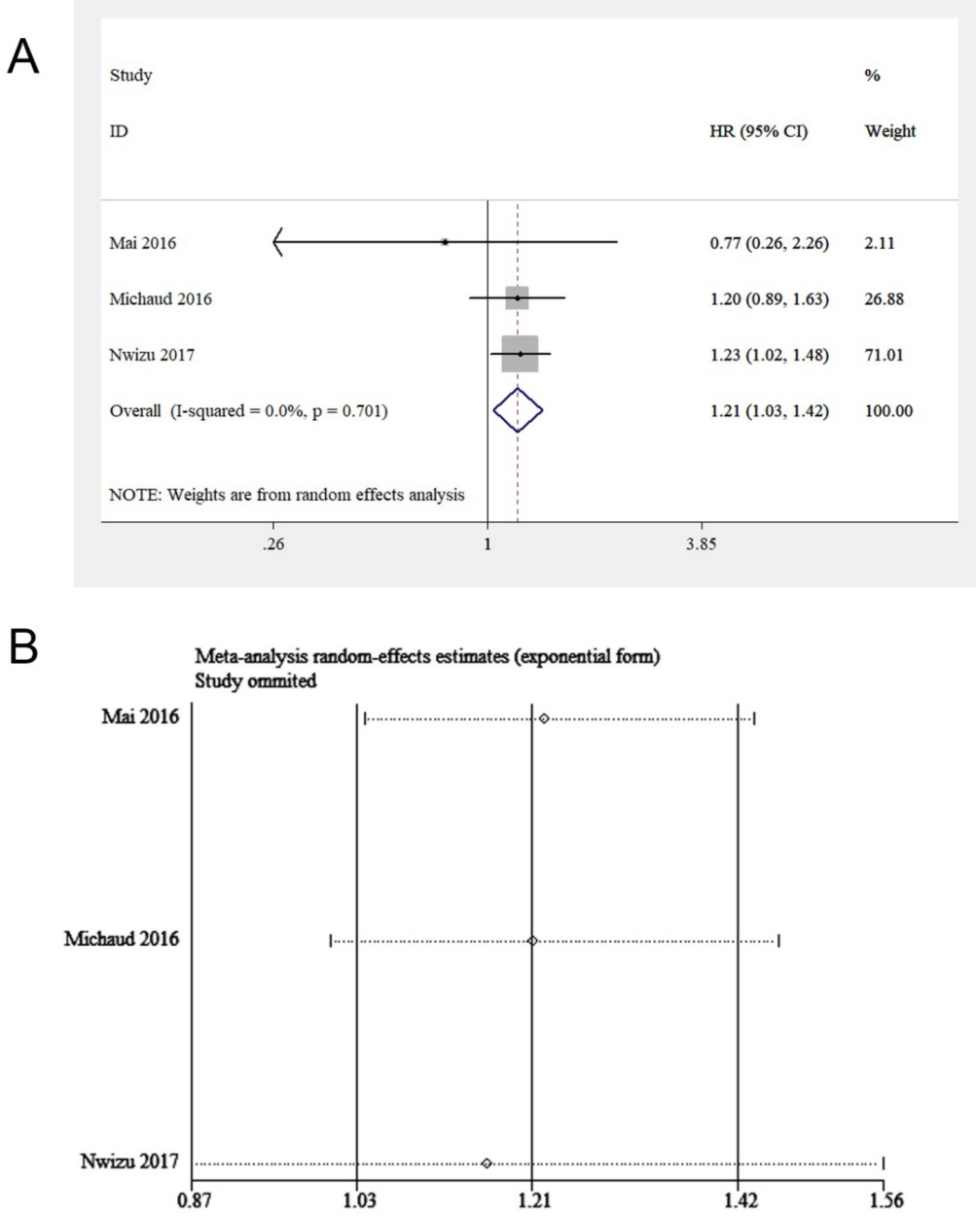
(**A**) Forest plot and (**B**) sensitivity analysis of studies on periodontitis and melanoma of the skin.

Three studies, including 87,139 participants, focused on the effect of periodontitis on melanoma of the skin 16, 18, 20. All of them adjusted important covariates, such as age, educational level and smoking status. By pooling these studies, a potential correlation between periodontitis and melanoma of the skin was discovered (HR = 1.21, 95% CI: 1.03-1.42, *I*^2^ = 0, *P* for heterogeneity = 0.70, **Figure 6A**). A sensitivity analysis was subsequently conducted. After excluding Nwizu's study, the overall estimate was no longer statistically significant (**Figure 6B**). The possible reason was that the sample size of this study accounted for a large part of the total sample size of the included studies. A subgroup analysis was not conducted because of the inclusion of insufficient studies.

## Discussion

A consensus about the correlation between periodontitis and five cancers (esophageal cancer, prostate cancer, kidney cancer, HM, and melanoma of the skin) was still lacking, highlighting the necessity for a meta-analysis. This meta-analysis, was performed on 8 studies (10 articles), with a remarkable sample size of more than 100,000 samples for most cancers. By pooling the included studies, a preliminary conclusion was drawn that participants with periodontitis might have enhanced risks of esophageal cancer, prostate cancer, HM, and melanoma of the skin with statistical significance, compared with those without periodontitis. However, no evident correlation between periodontitis and kidney cancer was found. The result of subgroup analysis showed that the periodontitis severity, duration of follow-up, age, sex and the number of participants might influence the result of overall analysis. In addition, observational studies with fewer participants might overestimate the actual correlation, compared with those with more participants. The correlations between periodontitis and two cancers (esophageal cancer and HM) were more significant among male participants than among female participants. Despite the confirmation of the correlation between periodontitis and HM, only the risk of NHL was found to be enhanced when studying the relationship between periodontitis and HM subtypes. The evidence of periodontitis enhancing the susceptibility to leukemia or MM was lacking, which might be attributed to the insufficiency of relevant studies. Therefore, more researches are needed to assess the correlations between periodontitis and HM subtypes.

At present, the exact mechanism of periodontitis promoting esophageal cancer, prostate cancer, HM and melanoma remains unclear. Among these cancers, the mechanisms underlying the relevance between periodontitis and malignant tumors in the esophagus and prostate were studied the most. In esophageal cancer tissues, periodontal microorganisms were found, including *P.gingivalis* 9, *T. denticola* 11 and *F. nucleatum* 10, which might induce inflammation of the esophagus. Cytokines secreted during inflammation, such as interleukin-1 (IL-1) and tumor necrosis factor-α (TNF-α), as well as reactive oxygen species (ROS), could activate cyclooxygenase 2 (COX-2), which contributed to the development, metastasis, and neo-angiogenesis of esophageal cancer 32. TNF-α and ROS led to the overexpression of glucose transporters (GLUT-1 and GLUT-4), which were responsible for the nourishment of tumor cells 32. Pathogens themselves were also related to esophageal carcinogenesis. *T. denticola* could trigger matrix metalloproteinases (MMP-8 and MMP-9) expressions 33, which correlated with immune regulation in tumor tissues 34. Moreover, an increased MMP-9 level was closely related to poor prognosis and metastasis of esophageal tumors 35. In addition, *P.gingivalis* could interfere with tumor suppressor p53 and promote carcinogenesis 36. As for prostate cancer, studies demonstrated a potential link between PSA levels and periodontal conditions 14. Moreover, periodontal microorganisms similar to those in subgingival plaque were found in prostate secretions 15. These studies supported the hypothesis that periodontal pathogens and inflammatory substances might migrate through the systemic circulation, causing inflammation of the prostate gland 37, 38. Local chronic inflammation led to hypoxia, contributing to the release of ROS. This caused COX to convert arachidonic acid into prostaglandins, which regulated cell proliferation 39. In addition, hypoxia stimulated vascular endothelial growth factor secretion, triggering neo-angiogenesis and fibroblast differentiation, which were promoters of prostatic hyperplasia or tumor 15. Other studies focused on mechanisms underlying the relevance between periodontitis and HM and melanoma. Studies showed that the citrullination of neutrophils and joint tissue proteins caused by periodontal pathogens (*P.gingivalis* and *Actinobacillus gingivalis*) might trigger autoimmune responses 40. Immune dysfunction, including autoimmune response and chronic inflammation, were risk factors for HM. Therefore, periodontitis might affect the long-term HM risk through autoimmune response or chronic inflammation 41. Regarding the relevance of periodontitis and cutaneous melanoma, it was discovered that the microbiome (including gut microbiome, oral microbiome and skin microbiome) had a significant impact on the development and immunotherapy of cutaneous melanoma 42-44. It was speculated that periodontitis might affect the balance of human microbiome and promote the development of melanoma.

However, the aforementioned findings were not sufficient to support a causal relationship between periodontitis and esophageal cancer, prostate cancer, HM and melanoma of the skin. More researches are needed to reveal the association between periodontitis and the aforementioned cancers.

Age, smoking and socioeconomic status are closely related to the development of cancers. Meanwhile, the aforementioned factors are also significantly related to periodontal conditions. Therefore, the possible influence of the aforementioned confounding factors should be addressed. All of the involved studies adjusted for age. Most studies adjusted for smoking and socioeconomic status. To address possible residual effects caused by smoking, three studies reported analyses restricted to never-smokers 17, 18, 26. When studying periodontitis and the risk of kidney cancer and melanoma of the skin, all of the included studies adjusted for smoking and socioeconomic status. As for periodontitis and the susceptibility to esophageal cancer, prostate cancer and HM, studies with adjustments of smoking and socioeconomic status were pooled in the subgroup analysis. The resulting HRs were statistically significant, indicating that the association between periodontitis and the aforementioned five cancers was independent of confounders such as age, smoking, and socioeconomic status.

No meta-analysis investigated whether periodontitis increased the risk of esophageal cancer, kidney cancer and melanoma of the skin. Corbella's meta-analysis reported a correlation between periodontitis and prostate tumor (involving 2 studies and 63,708 samples) 45. Compared with Corbella's meta-analysis, the present meta-analysis had more advantages. First, the results were obtained from a larger database of 4 studies with 229,256 participants. Besides, the present meta-analysis investigated the effect of important confounders in subgroup analysis, such as age, smoking status, socioeconomic status, sample size and periodontitis severity, while Corbella *et al.* performed no such investigations. Similarly, Wu's meta-analysis recently reported a link between periodontitis and HM. 46 However, Wu *et al.* did not address the possible influence of some significant confounders, such as age and socioeconomic status, which were the risk factors for both periodontitis and cancers. Moreover, they did not calculate the respective risk of leukemia, NHL and MM for patients with periodontitis. On the contrary, the present meta-analysis carefully discussed covariates including age, socioeconomic status, follow-up years, research quality and sample size, in the subgroup analysis. More importantly, it further evaluated the correlation between periodontitis and HM sub-types, including leukemia, lymphoma and MM.

Nevertheless, this study had certain limitations. First, due to the inadequate number of involved literatures, the results obtained were not robust enough. Therefore, it was tentatively suggested that the relevance between periodontitis and the aforementioned cancers was a potential link, not a clear correlation. Second, the confirmation on periodontitis varied from study to study. A majority of included studies were based on standardized dental examinations, while two studies confirming periodontitis based on self-reported tooth mobility 18, 27. However, LaMonte *et al.* 47 assessed the accuracy of self-report. The specificity of self-reporting was up to 94% 48. Third, the relevance between periodontitis severity and cancers remained unclear. Some studies were carefully grouped participants according to the severity of periodontitis, but their grouping criteria were not consistent. For each type of cancer, the number of studies stratified by the severity of periodontitis was still insufficient, which might explain why the HRs obtained in the subgroup analysis of periodontitis severity and risk of HM was not statistically significant. Fourth, in subgroup analysis, some of the 95%CIs of *I^2^* were not reported. This was because 95%CIs of *I^2^* cannot be calculated when the number of studies included was insufficient or the value of *Chi^2^* for heterogeneity was too small 30. Fifth, for kidney cancer, the heterogeneity was significant, which was probably attributed to the insufficiency of included articles. With only two articles involved, the statistical effectiveness of overall estimates for kidney cancer was lacking. Sixth, when assessing the relationship between periodontitis and melanoma of the skin, the exclusion of a study conducted by Nwizu *et al.* caused a great change in the result 18, which might be attributed to the large sample size of this study and the insufficiency of the number of included studies. Finally, further exploration of the correlation between periodontitis and cancer sub-types was restricted due to insufficient data.

## Conclusions

The present meta-analysis of recent epidemiological studies supported that periodontitis was potentially associated with the development of esophageal cancer, prostate cancer, HM, and melanoma of the skin, highlighting the significance of early prevention and treatment of periodontitis. It must be noted that this meta-analysis was not sufficient to support a causal relationship between periodontitis and the aforementioned cancers. It only revealed a potential link. Moreover, the exact mechanism underlying this potential link remained unclear. Multi-center observational studies with an adequate number of participants and multivariable adjustments are required to further assess the correlation between periodontitis and cancers.

## Supplementary Material

Supplementary figures.

## Figures and Tables

**Table 1:** Classification of periodontitis severity

	Periodontitis Severity	
Classification criteria*	Mild/Moderate	Severe
AL or/and PD^25^, ^26^	at least two teeth with interproximal AL ≥4 mm or at least two teeth with interproximal PD ≥5 mm	at least two teeth with interproximal AL ≥6 mm and at least one tooth with interproximal PD ≥5 mm
AL^26^	>10% to <30% of examined sites (six sites on all teeth) having AL>3 mm	>30% of examined sites (six sites on all teeth) with AL>3 mm
ACH or/and tooth loss^20^	whole mouth mean ACH ≥2mm to <3mm, or at least one site ACH ≥4mm, and no tooth loss due to periodontitis	whole mouth mean ACH ≥ 3mm, or at least two sites ACH ≥5mm, or tooth loss due to periodontitis

AL: attachment loss; ACH: alveolar crestal height; PD: probing depth; *It can be considered as mild/moderate periodontitis by meeting any one of the criteria of mild/moderate. The same is true for severe.

**Table 2:** Summary of the enrolled studies

Research ID	Author year	Origin	Study design	Mean follow-up period (yrs)	Mean age	Sex	Sample size	Periodontitis assessment	Severity of periodontitis	Cancer assessment	Cancer types	NOS scores
#1	Chung 2016^21^	China	Retrospective cohort	5	54.1	Both	80,280	At least two consensus diagnoses	Unspecified	Ambulatory care claims code	Hematological malignancy, etc.	7
	Lee 2014^24^	China	Retrospective cohort	8.425	39.8	Both	718,409	At least two consensus diagnoses	Dental prophylaxis group; Intensive treatment group^a^; PD without treatment group.	Ambulatory care claims code	Esophagus, etc.	7
#2	Nwizu 2017^18^	America	Prospective cohort	8.32	68.3	Female	65,869	Self-report	Unspecified	Medical records	Esophagus, Kidney, Hematological malignancy, Melanoma of the skin, etc.	6
#3	Michaud 2018^26^	America	Prospective cohort	14.7	62.5	Both	6,056	Clinical diagnosis	No/mild; Moderate; Severe	State cancer registries	Prostate, Hematological malignancy, etc.	8
#4	Mai 2016^20^	America	Prospective cohort	12.2±4.2	66.7	Female	1,337	Defined by alveolar crestal height	Mild/ moderate; Severe	Medical records	Melanoma of the skin, Hematological malignancy, etc.	7
#5	Michaud 2008^17^	America	Prospective cohort	17.7	54.42	Male	48,375	Radiographs for bone loss assessment	Unspecified	Medical records or pathology reports	Esophagus, Kidney, Hematological malignancy, etc.	7
	Michaud 2016^16^	America	Prospective cohort	26	52.89	Male	19,933	Radiographs for bone loss assessment	Unspecified	Medical records or pathology reports	Prostate, Melanoma of the skin, etc.	7
#6	Arora 2010^27^	Sweden	Prospective cohort	27	51	Both	15,333	Self-report teeth mobility	Minor mobility; Periodontal disease	Records in cancer register	Prostate, etc.	7
#7	Lee 2017^28^	South Korea	Retrospective cohort	12	61.2	Both	187,934	Clinical and radiographic diagnoses	Unspecified	diagnostic codes	Prostate, etc.	6
#8	Ahn 2012^25^	America	Prospective cohort	unreported	38.99	Both	12,605	Periodontal attachment loss and pocket depth	Unspecified	Mortality File	Esophagus, etc.	6

CI: confidence interval; NOS: Newcastle-Ottawa scale; ^a^ For example, subgingival scaling, gingivectomy, periodontal flap surgery.

**Table 3:** Adjusted covariates in studies included in the meta-analysis

Study	Adjusted covariates
Chung 2016	Age, sex, urbanization, salary, location
Lee 2014	Age, sex, drinking status, comorbidities of esophageal malignancy
Nwizu 2017	Age, education, geographic location, tobacco consumption, family history of malignant tumors, exercise habit, secondhand cigarette smoke, drinking status, fruit and vegetable intake
Michaud 2018	Age, location, educational status, alcohol consumption, blood glucose level, BMI, race, smoking behaviors
Mai 2016	Age, oral hygiene habits, family history of malignant tumors, educational status, tobacco consumption, secondhand cigarette smoke, body mass index
Michaud 2008	Age, frequency of exercise, BMI, energy intake, dietary collocation, drinking status, tobacco consumption, blood glucose level, smoking behaviors, calcium supplement, residence, race
Michaud 2016	Age, drinking habit, tobacco consumption, height, frequency of exercise, BMI, residence region
Arora 2010	Age, gender, blood glucose level, tobacco consumption, tobacco consumption of partner, drinking behavior, BMI, vocation, education
Lee 2017	Age, sex, family economic status, location, cardiovascular and cerebrovascular diseases, physical activity, drinking habit, tobacco consumption
Ahn 2012	Sex, age , ethnic origin, smoking status, education

BMI: Body mass index.

**Table 4:** Results of overall estimates

Cancer	No. of studies	Heterogeneity		Model	Meta-analysis		
		*I^2^* (95% CI)	*p*		HR	95% CI	*p*
Esophageal	4	60% (0%-87%)	0.06	Random	1.79	1.15-2.79	0.009
Prostate	4	0% (0%-85%)	0.60	Random	1.20	1.09-1.31	0.0002
Kidney	2	44% (0%-84%)	0.18	Random	1.30	0.96-1.76	0.09
Hematological	5	0% (0%-79%)	0.61	Random	1.19	1.09-1.29	<0.0001
Melanoma of the skin	3	0% (0%-90%)	0.70	Random	1.21	1.03-1.42	0.02

No. of studies: the number of studies.

**Table 5:** Subgroup analysis of studies on periodontitis and esophageal cancer

Subgroup	No. of studies	Heterogeneity		Model	Meta-analysis		
		*I^2^* (95%CI)	*p*		HR	95%CI	*p*
Overall analysis	4	60% (0%-87%)	0.06	Random	1.79	1.15-2.79	0.009
**Research quality**							
Medium	2	0%	0.85	Random	3.37	1.83-6.22	<0.0001
High	2	0%	0.65	Random	1.35	1.04-1.75	0.03
**Mean age**							
<50	2	59% (0%-90%)	0.12	Random	1.81	0.67-4.88	0.24
≥50	2	71%	0.06	Random	2.12	1.01-4.45	0.05
**Sex**							
Male	2	0%	0.88	Random	1.41	1.08-1.83	0.01
Female	2	75% (0%-94%)	0.05	Random	1.37	0.17-11.02	0.77
**Adjusted covariates**							
With smoking status	3	63% (0%-89%)	0.07	Random	2.28	1.16-4.49	0.02
With socio-economic status	2	0%	0.85	Random	3.37	1.83-6.22	<0.0001

No. of studies: the number of studies.

**Table 6:** Subgroup analysis of studies on periodontitis and prostate cancer

Subgroup	No. of studies	Heterogeneity		Model	Meta-analysis		
		*I^2^* (95%CI)	*p*		HR	95%CI	*p*
Overall analysis	4	0% (0%-85%)	0.60	Random	1.20	1.09-1.31	0.0002
Periodontitis severity							
Mild/moderate	2	0%	0.41	Random	1.14	0.95-1.37	0.17
Severe	2	0%	0.48	Random	1.34	1.06-1.68	0.01
Adjusted covariates							
With smoking status	4	0% (0%-85%)	0.60	Random	1.20	1.09-1.31	0.002
With socio-economic status	3	0% (0%-58%)	0.40	Random	1.20	1.08-1.33	0.0007
Mean age							
≤60	2	11% (0%-43%)	0.29	Random	1.26	1.03-1.54	0.02
>60	2	0%	0.53	Random	1.18	1.05-1.31	0.001
Sample size							
≤15000	2	0%	0.42	Random	1.30	1.09-1.55	0.004
>15000	2	0%	0.87	Random	1.16	1.03-1.29	0.01

No. of studies: the number of studies.

**Table 7:** Subgroup analysis of studies on periodontitis and HM

Subgroup	No. of studies	Heterogeneity		Model	Meta-analysis		
		*I^2^* (95%CI)	*p*		HR	95%CI	*p*
Overall analysis	5	0% (0%-79%)	0.61	Random	1.19	1.09-1.29	<0.0001
**Mean age**							
<60	2	0%	0.38	Random	1.23	1.11-1.37	0.0001
≥60	3	0% (0%-90%)	0.75	Random	1.11	0.96-1.28	0.15
**Sex**							
Male	2	0%	0.38	Random	1.33	1.14-1.54	0.0003
Female	3	0% (0%-90%)	0.51	Random	1.09	0.93-1.27	0.27
**Periodontitis severity**							
Mild/moderate	2	0%	0.45	Random	1.08	071-1.65	0.73
Severe	2	0%	0.50	Random	1.47	0.92-2.33	0.11
**Adjusted covariates**							
With smoking status	4	0% (0%-85%)	0.45	Random	1.19	1.07-1.33	0.001
With socio-economic status	4	0% (0%-85%)	0.82	Random	1.14	1.03-1.27	0.009
**Smoking status**							
Non-smokers	3	0% (0%-90%)	0.63	Random	1.30	1.14-1.48	0.0001
**Follow-up years**							
≤10	2	0%	0.42	Random	1.13	1.02-1.26	0.02
>10	3	0% (0%-90%)	0.96	Random	1.29	1.12-1.48	0.0004
**Sample size**							
<10000	2	0%	1.00	Random	1.24	0.91-1.69	0.18
≥10000	3	23% (0%-70%)	0.27	Random	1.18	1.07-1.31	0.001
**Hematologic cancers sub-types**							
Leukemia	2	0%	0.38	Random	1.21	0.98-1.49	0.08
Non-Hodgkin lymphoma	2	44% (0%-85%)	0.21	Random	1.20	1.01-1.43	0.04
Multiple myeloma	2	0%	0.44	Random	1.17	0.89-1.54	0.27

No. of studies: the number of studies.

## Abbreviations

ACH: Alveolar crestal height
AL: attachment loss
BMI: body mass index
CI: confidence interval
COX: cyclooxygenase
HM: hematological malignancy
HR: hazard ratio
MM: multiple myeloma
NHL: non-Hodgkin's lymphoma
NOS: Newcastle-Ottawa Scale
PD: probing depth
VEGF: vascular endothelial growth factor
ROS: reactive oxygen species
