# Supplementary material for: Potential risk of certain cancers among patients with Periodontitis: a supplementary meta-analysis of a large-scale population
Source: Int J Med Sci. 2020 Sep 12;17(16):2531–43. doi: 10.7150/ijms.46812 (PMC7532473; doi:10.7150/ijms.46812)
Supplement: Supplementary file 1 — Supplementary figures. [file ijmsv17p2531s1.pdf]

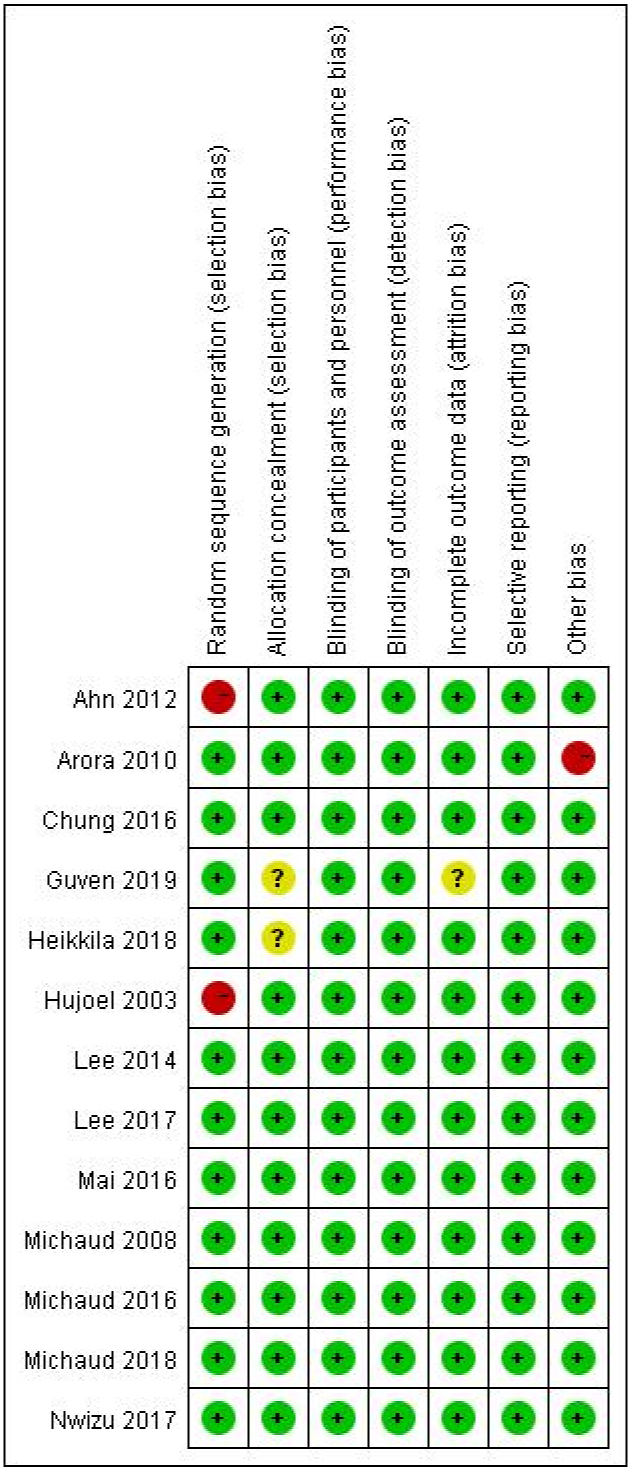

Figure S1. Risk of bias summary: each risk of bias item for each included studies.

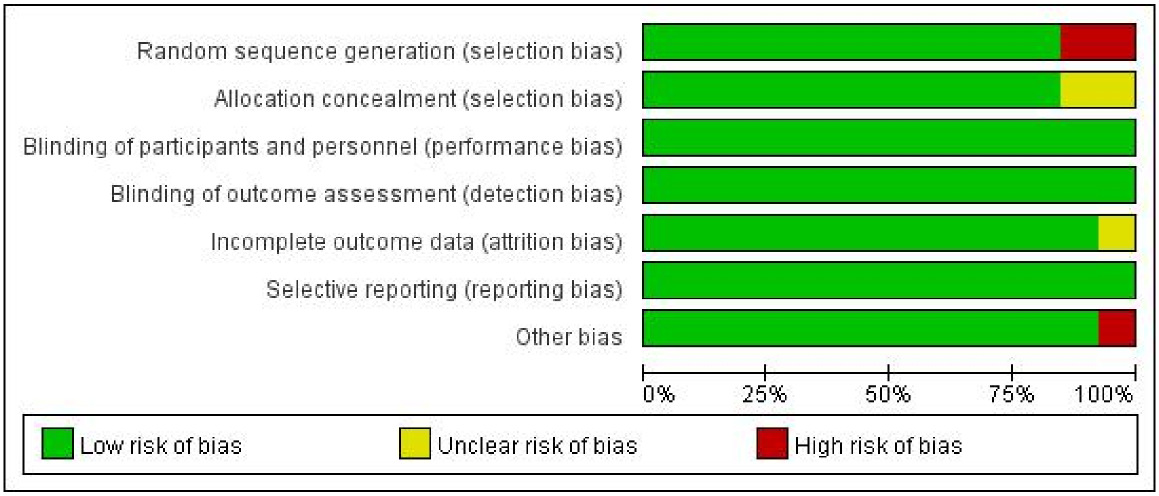

Figure S2. Risk of bias graph: each risk of bias item presented as percentages across all included studies.
